# Study of efficacy and antibody duration to fourth-dose booster of Ad5-nCoV or inactivated SARS-CoV-2 vaccine in Chinese adults: a prospective cohort study

**DOI:** 10.3389/fimmu.2023.1244373

**Published:** 2023-09-06

**Authors:** Nani Xu, Yu Xu, Rongrong Dai, Lin Zheng, Pan Qin, Peng Wan, Yejing Yang, Jianmin Jiang, Hangjie Zhang, Xiaowei Hu, Huakun Lv

**Affiliations:** ^1^ Department of Immunization Program, Xihu District Center for Disease Control and Prevention, Hangzhou, China; ^2^ Department of Vaccine, Clinical Trials, CanSino Biologics, Tianjin, China; ^3^ School of Public Health, Hangzhou Medical College, Hangzhou, China; ^4^ Department of Immunization Program, Zhejiang Provincial Center for Disease Control and Prevention, Hangzhou, China; ^5^ Key Lab of Vaccine, Prevention and Control of Infectious Disease of Zhejiang Province, Zhejiang Provincial Center for Disease Control and Prevention, Hangzhou, China

**Keywords:** SARS-CoV-2, COVID-19, fourth dose, Ad5-nCoV, breakthrough infection

## Abstract

**Introduction:**

China experienced a record surge of coronavirus disease 2019 cases in December 2022, during the pandemic.

**Methods:**

We conducted a randomized, parallel-controlled prospective cohort study to evaluate efficacy and antibody duration after a fourth-dose booster with Ad5-nCoV or inactivated severe acute respiratory syndrome coronavirus 2 (SARS-CoV-2) vaccine.

**Results:**

A total of 191 participants aged ≥18 years who had completed a three-dose regimen of the inactivated SARS-CoV-2 vaccine 6 months earlier were recruited to receive the intramuscular Ad5-nCoV booster or the inactivated SARS-CoV-2 vaccine. The Ad5-nCoV group had significantly higher antibody levels compared with the inactivated vaccine group at 6 months after the fourth vaccination dose. After the pandemic, the breakthrough infection rate for the Ad5-nCoV and the inactivated vaccine groups was 77.89% and 78.13%, respectively. Survival curve analysis (*p* = 0.872) and multivariable logistic regression analysis (*p* = 0.956) showed no statistically significant differences in breakthrough infection between the two groups.

**Discussion:**

Compared with a homologous fourth dose, a heterologous fourth dose of Ad5-nCoV elicited a higher immunogenic response in healthy adults who had been immunized with three doses of inactivated vaccine. Nevertheless, the efficacy of the two vaccine types was equivalent after the pandemic.

## 1 Introduction

On 30 January 2020, the World Health Organization declared that the coronavirus disease 2019 (COVID-19) outbreak constituted a public health emergency of international concern (1). To date, 768.0 million confirmed cases and 6.9 million deaths have been recorded globally (2). Despite the administration of 13.39 billion vaccine doses (2), the COVID-19 epidemic has not yet resolved. On 27 January 2023, the World Health Organization determined that the ongoing COVID-19 pandemic continued to constitute a public health emergency of international concern (3). Emerging variants of severe acute respiratory syndrome coronavirus 2 (SARS-CoV-2) that are capable of escaping an immune attack have reduced the protection conferred by vaccines (4–7). In addition, the effectiveness of the vaccine, which included a two-dose regimen and a booster (third dose) vaccine, has declined over time (8–11).

Studies in Canada, Singapore, and other countries have shown that a second booster immunization provides additional protection against COVID-19 and reduces severe illness and death (12–16). More than 71.7% of the Chinese population has been immunized with three doses of inactivated SARS-CoV-2 vaccine (17). Previous studies in China have shown that the efficacy of the first booster dose of Ad5-nCoV (heterologous booster) is superior to that of inactivated vaccine (homologous booster) (18, 19). Furthermore, a heterologous fourth dose with either aerosolized Ad5-nCoV or intramuscular Ad5-nCoV was safe and highly immunogenic in healthy adults who were previously immunized with three doses of the CoronaVac vaccine within 28 days (20). However, antibody levels following a fourth dose of Ad5-nCoV at 6 months after vaccination with three doses of inactivated SARS-CoV-2 vaccine have not yet been assessed.

On 7 December 2022, the Chinese government issued the “New ten measures” as an adjustment to its “Twenty measures” COVID-19 prevention and control policy released on 11 November 2022 (21). As a result, the COVID-19 epidemic in China changed dramatically. On 1 February 2023, the Chinese Center for Disease Control and Prevention reported an overall infection rate of 87.54% based on data reported between 9 December 2022 and 30 January 2023 (22, 23). In this context, assessing the real-world breakthrough infection rate after the fourth dose of immunization with Ad5-nCoV or inactivated SARS-CoV-2 vaccine is important.

This parallel-controlled prospective cohort study aimed to investigate antibody levels and breakthrough infection after a fourth immunization dose of Ad5-nCoV or inactivated SARS-CoV-2 vaccine.

## 2 Materials and methods

### 2.1 Study design and participants

A single-center, randomized, parallel-controlled prospective cohort study of a second booster dose (or fourth vaccine dose) with a heterologous booster (Ad5-nCoV) or a homologous booster (inactivated SARS-CoV-2 vaccine) was conducted in Xihu District, Hangzhou City, Zhejiang Province, China. Participants aged ≥18 years with stable medical conditions who had completed a three-dose regime of the inactivated SARS-CoV-2 vaccine (CoronaVac or Covilo) 6 months earlier were recruited from the community.

A screening visit before the participants were enrolled allowed exclusion of the following conditions: history of infection with SARS-CoV-2; pregnant or lactating; use of immunosuppressives; fever; history of severe anaphylaxis to vaccines; severe and/or uncontrolled respiratory disease and cardiovascular disease; hypertension (systolic pressure ≥ 180 mmHg/diastolic pressure ≥ 110 mmHg); diabetes; neurologic illness; and other underlying diseases that could interfere with the evaluation of the primary study endpoints. Previous SARS-CoV-2 infection history was confirmed with participant recall and by checking participants’ recent medical visits.

The Research Ethics Committee of the Zhejiang Provincial Center of Disease Control and Prevention reviewed and approved the study protocol. The informed consent form was signed by all participants before enrolment.

### 2.2 Procedures

In May 2022, 200 eligible participants were randomly assigned to receive one dose of Ad5-nCoV (Convidecia, 0.5 mL of 5 × 10^10^) viral particles) or inactivated vaccine (CoronaVac or Covilo, 0.5 mL) via intramuscular injection. Randomization was conducted using a sealed envelope system in which the number associated with each vaccine group was displayed at a 1:1 ratio. The type of inactivated vaccine administered as the fourth dose was matched to the vaccine type each participant had already received as a first booster dose (or third dose).

Blood samples were collected from each participant at baseline, before participants received the fourth dose of vaccination and again 6 months later, in December 2022. A commercial anti–SARS-CoV-2 RBD IgG ELISA kit (Vazyme Medical Technology, Nanjing, China) was used to measure the wild-type SARS-CoV-2 Receptor Binding Domain (RBD)-specific IgG response. The Reed–Muench method was also used to assess the levels of neutralizing antibody against the Omicron BA.4/5 subvariant using a pseudovirus-neutralization test, which consisted of a vesicular stomatitis virus pseudovirus system that expresses the spike glycoprotein (50% neutralization titer).

In March 2023, after the pandemic and approximately 10 months after the fourth vaccination dose, the participants were followed up by telephone to collect information on breakthrough infection with SARS-CoV-2.

### 2.3 Statistical analysis

The level of antibodies was presented as the geometric mean titer (GMT) and geometric mean fold increase (GMFI). The 95% confidence interval (CI) was calculated on the basis of the t-distribution of the log-transformed values back-transformed to the original scale. The Student’s t-test, Chi-squared test, or Fisher’s exact test was applied to analyze categorical data. A Cox model was used to estimate the cumulative probability of breakthrough infection. A multivariable logistic regression analysis was performed to test the adjusted association between the main independent variable, which was defined as the type (inactivated vaccine or Ad5-nCoV) of the fourth-dose vaccine, and 1) the seropositivity rates of anti-RBD IgG and neutralizing antibodies against the BA.4/5 pseudovirus and 2) the risk of breakthrough infection with SARS-CoV-2. *P*-values of less than 0.05 were considered statistically significant. All statistical analyses were conducted using SPSS Statistics (IBM Corporation, Armonk, NY, USA) and GraphPad Prism 9 (San Diego, CA, USA).

## 3 Results

### 3.1 Study participants

**Figure 1:**
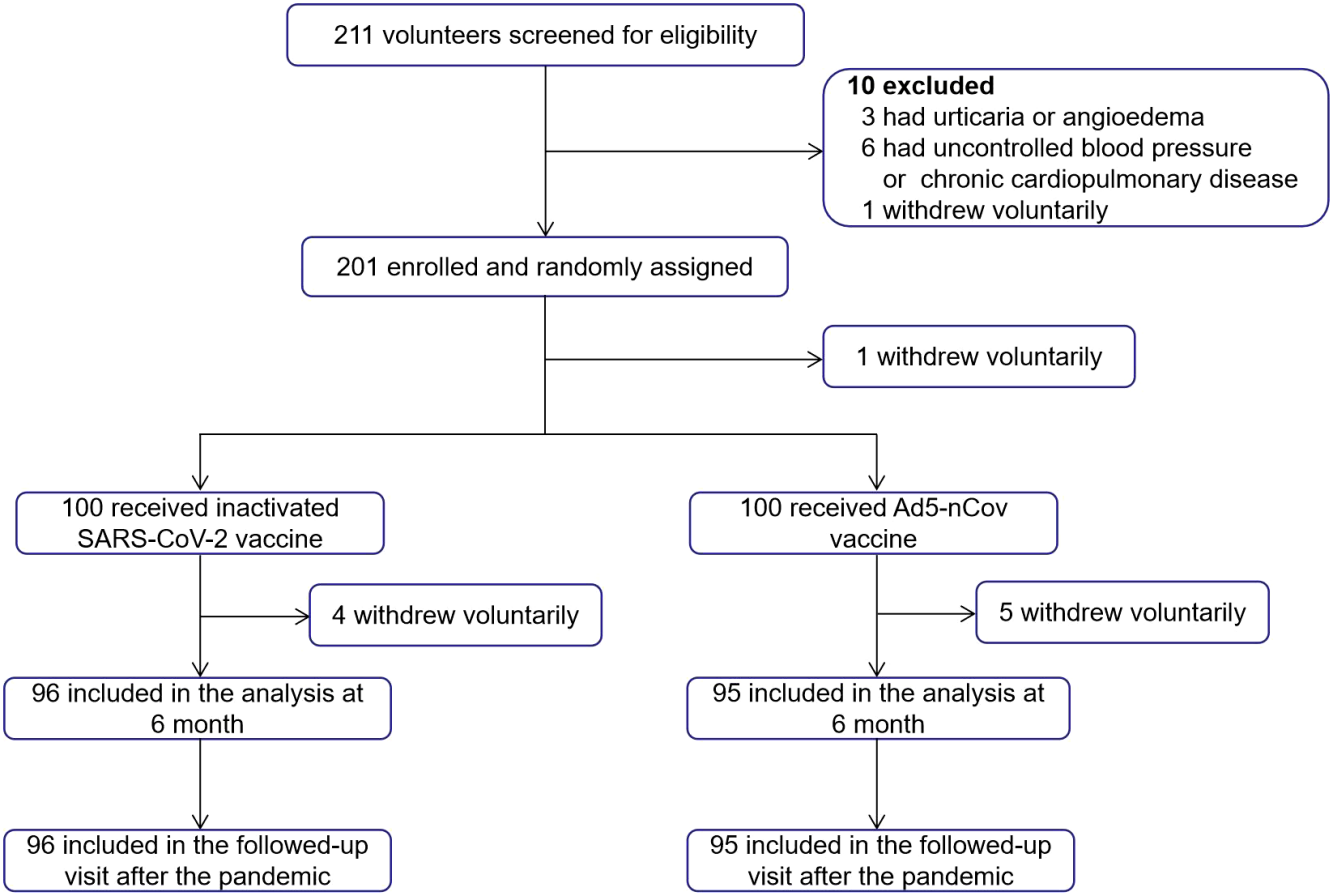
Trial profile. A total of 211 volunteers were recruited and screened for eligibility. Overall, 201 participants were enrolled and randomly assigned, 200 participants received the booster dose vaccination, and one participant withdrew voluntarily after randomization. Participants in each group (n = 96 and n = 95) completed both the planned visits at 6 months after vaccination and the telephone follow-up in March 2023.

In total, 211 volunteers aged ≥18 years who had received three doses of inactivated vaccine (CoronaVac or Covilo) ≥6 months earlier were recruited and screened for eligibility in May 2022. A total of 201 participants were sequentially enrolled and randomly assigned to two groups. One participant withdrew voluntarily after randomization. In total, 200 participants received a fourth dose of Convidecia (treatment group, heterologous booster dose, n = 100) or CoronaVac/Covilo (control group, homologous booster dose, n = 100). Finally, 95 (treatment group) and 96 (control group) participants completed both the follow-up at 6 months after vaccination and the telephone follow-up in March 2023, after the pandemic (**Figure 1**).

Among the 191 participants who ultimately completed the follow-up, 87 (45.55%) were female and 56 (29.32%) had underlying chronic diseases. The mean patient age was 50.52 (standard deviation, 15.37) years. The mean time interval since the last booter dose of inactivated vaccine was 6.13 (standard deviation, 0.04) months. The baseline characteristics of the two vaccine groups were comparable (**Table 1**).

**Table 1:** Comparison of baseline characteristics between the two vaccine groups.

Variable	Inactivated vaccine (n = 96)	Ad5-nCoV (n = 95)	*p*
Gender			
Male	54 (56.25)	50 (52.63)	0.616
Female	42 (43.75)	45 (47.37)	
Age, years			
18–59	56 (58.33)	56 (58.95)	0.931
≥60	40 (41.67)	39 (41.05)	
Mean age (SD)	50.43 (15.77)	50.62 (15.05)	0.903^b^
BMI (kg/m^2^)			
<18.5	1 (1.04)	4 (4.21)	0.698^a^
18.5–23.9	54 (56.25)	45 (47.43)	
24.0–27.9	30 (31.25)	33 (34.74)	
≥28.0	11 (11.46)	13 (13.68)	
Exercise			
≥3 h per week	62 (64.58)	68 (71.58)	0.300
<3 h per week	34 (35.42)	27 (28.42)	
Sleep			
Regular	74 (77.08)	69 (72.63)	0.478
Have insomnia	22 (22.92)	26 (27.37)	
Underlying chronic diseases			
Yes	25 (26.04)	31 (32.63)	0.317
No	71 (73.96)	64 (67.37)	
Vaccination history			
Influenza			
Yes	40 (41.67)	45 (47.37)	0.428
No	56 (58.33)	50 (52.63)	
Mumps			
Yes	2 (2.08)	3 (3.16)	0.683^a^
No	94 (97.92)	92 (96.84)	
Rabies			
Yes	15 (15.63)	21 (22.11)	0.252
No	81 (84.38)	74 (77.08)	
Time interval since the last priming dose of inactivated vaccine, months			
Mean (SD)	6.13 (0.04)	6.13 (0.05)	0.994^b^

Influenza vaccination history within 1 year before the second booster dose of Ad5-nCoV or inactivated SARS-CoV-2 vaccine was collected; mumps and rabies vaccination history were collect without time limitation. Data are number of participants (%), mean (SD), or median (IQR). Underlying chronic diseases included cardiovascular and cerebrovascular diseases, hypertension, and chronic obstructive pulmonary disease. The comparison was analyzed using the Chi-squared test unless marked as ^a^Fisher’s exact test or ^b^Student’s t-test. SD, standard deviation; IQR, interquartile range.

### 3.2 Immunogenicity assessment

#### 3.2.1 SARS-CoV-2 antibody levels in the two vaccine groups

**Figure 2:**
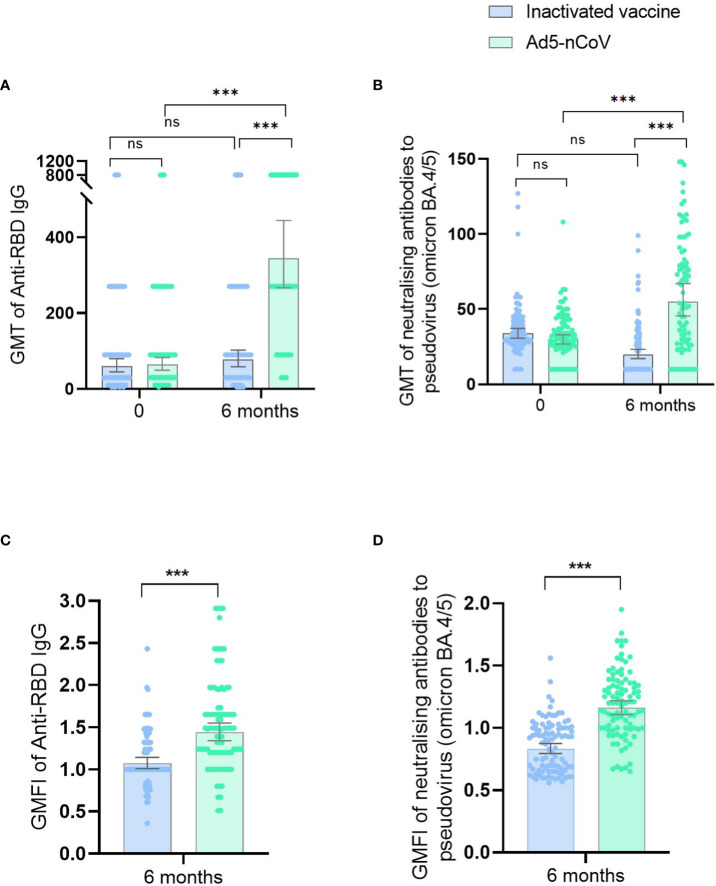
Antibody responses before and after booster vaccination. ID50 of SARS-CoV-2 RBD-specific IgG antibodies **(A)** and ID50 of pseudovirus-neutralizing antibodies against Omicron BA.4/5 **(B)** on day 0 (before booster vaccination) and at 6 months after booster vaccination in the inactivated SARS-CoV-2 vaccine group and the Ad5-nCoV group. ID50 of SARS-CoV-2 RBD-specific IgG antibodies **(C)** and ID50 of pseudovirus-neutralizing antibodies against Omicron BA.4/5 **(D)** at 6 months after booster vaccination in the inactivated SARS-CoV-2 vaccine group and the Ad5-nCoV group with different age subgroups. GMFIs of SARS-CoV-2 RBD-specific IgG antibodies **(E)** and GMFIs of pseudovirus-neutralizing antibodies against Omicron BA.4/5 **(F)** at 6 months after booster vaccination in the inactivated SARS-CoV-2 vaccine group and the Ad5-nCoV group. ns, no significance; ^*^ *P* < 0.05; ^**^ *P* < 0.01; ^***^ *P* < 0.001.

At enrollment and before receiving the second vaccine booster, the average GMT of anti-RBD IgG in the 191 participants was 61.78, and the GMT of neutralizing antibodies against the BA.4/5 pseudovirus was 31.68. No statistical difference was observed in the baseline antibody levels between the inactivated vaccine group and the Ad5-nCoV group (**Figure 2**).

Six months after the fourth vaccination dose, anti-RBD IgG GMTs were significantly higher in the Ad5-nCoV group (344.22; 95% CI, 266.65–444.35) than in the inactivated vaccine group (77.10; 95% CI, 58.24–102.05; *p* < 0.001). Similarly, the GMFI was higher for the Ad5-nCoV group (1.53) than that for the inactivated vaccine group (1.13; *p* < 0.001). GMTs for the neutralizing antibodies against the BA.4/5 pseudovirus were 55.26 (95% CI, 45.52–67.08) and 20.03(95% CI, 17.23–23.28) for the treatment and control groups, respectively (*p* < 0.001), and the GMFIs were 1.19 and 0.86 for the treatment and control groups, respectively (*p* < 0.001; **Supplementary Table 1**; **Figure 2**).

Compared with the baseline antibody levels before the fourth vaccination dose, no significant difference in the GMT of anti-RBD IgG was observed at 6 months after the fourth-dose vaccination in the inactivated vaccine group (*p* = 0.212); however, the GMT of neutralizing antibodies against the BA.4/5 pseudovirus was lower versus baseline (*p* < 0.001). In contrast, significantly higher GMTs of anti-RBD IgG (*p* < 0.001) and neutralizing antibodies against the BA.4/5 pseudovirus (*p* < 0.001) were observed compared with pre-immunization levels in the Ad5-nCoV group (**Figure 2**).

#### 3.2.2 Factors influencing antibody duration

Compared with their younger peers, participants ≥60 years had significantly lower GMTs of anti-RBD IgG (*p* = 0.001) and neutralizing antibodies against the BA.4/5 pseudovirus (*p* = 0.015) in the inactivated vaccine group and lower GMTs of neutralizing antibodies against the BA.4/5 pseudovirus (*p* = 0.018) in the Ad5-nCoV group (**Table 2**; **Figure 2**). In the inactivated vaccine group, persons with underlying chronic diseases had lower GMTs of anti-RBD IgG (*p* = 0.033) compared with those without diseases (**Table 2**).

**Table 2:** Associations between participants’ characteristics and antibody GMT.

Variable	Anti–RBD-IgG				Neutralizing antibodies to pseudovirus (BA.4/5)			
	Inactivated vaccine (n = 96)		Ad5-nCov (n = 95)		Inactivated vaccine (n = 96)		Ad5-nCov (n = 95)	
	GMT	*p*	GMT	*p*	GMT	*p*	GMT	*p*
**Gender**		0.393		0.714		0.055		0.177
Male	65.14 (44.06, 96.30)		329.04 (229.41, 471.93)		17.62 (14.68, 21.16)		51.04 (38.6, 67.48)	
Female	95.75 (63.77, 143.77)		361.91 (248.73, 526.58)		23.62 (18.37, 30.36)		60.36 (45.81, 79.54)	
**Age, years**		0.001*		0.3007		0.015*		0.018*
18–59	101.98 (70.11, 148.33)		384.35 (270.62, 545.88)		23.37 (18.84, 28.98)		72.23 (57.08, 91.42)	
≥60	52.11 (34.62, 78.46)		293.81 (201.36, 428.70)		16.14 (13.35, 19.52)		37.62 (27.84, 50.83)	
**BMI (kg/m^2^)**		0.079^a^		0.443^a^		0.356^a^		0.833^a^
<18.5	89.13 (89.13, 89.13)		467.65 (81.42, 2686.15)		10.00 (10.00, 10.00)		95.52 (63.3, 144.15)	
18.5–23.9	83.59 (56.59, 123.47)		344.66 (239.29, 496.44)		22.09 (17.62, 27.69)		60.94 (47.67, 77.90)	
24.0–27.9	75.24 (43.22, 130.98)		279.14 (179.64, 433.76)		17.01 (13.54, 21.38)		40.26 (28.38, 57.10)	
≥28.0	54.62 (29.82, 100.06)		530.86 (220.22, 1279.64)		20.60 (13.86, 30.63)		74.36 (35.24, 156.91)	
**Exercise**		0.081		0.325		0.080		0.150
≥3 h per week	66.28 (48.06, 91.42)		372.99 (281.99, 493.36)		18.15 (15.31, 21.52)		49.63 (39.60, 62.22)	
<3 h per week	101.55 (58.95, 174.95)		281.21 (156.81, 504.31)		23.96 (17.86, 32.16)		72.43 (49.55, 105.87)	
**Sleep**		0.235		0.292		0.922		0.779
Regular	75.43 (55.76, 102.04)		316.60 (228.96, 437.79)		19.95 (17.08, 23.30)		51.45 (40.62, 65.17)	
Have insomnia	82.96 (39.78, 172.99)		429.76 (293.86, 628.51)		20.31 (13.23, 31.17)		66.79 (47.42, 94.08)	
**Underlying chronic diseases**		<0.001*		0.190		0.394		0.930
Yes	33.55 (23.91, 47.06)		270.00 (178.08, 409.37)		17.95 (13.83, 23.30)		75.49 (46.52, 122.51)	
No	70.37 (56.58, 87.53)		387.19 (279.88, 535.63)		20.82 (17.31, 25.03)		77.67 (54.99, 109.70)	
Vaccination history								
**Influenza**		0.415		0.851		0.848		0.845
Yes	67.21 (44.78, 100.88)		353.18 (244.76, 509.62)		19.68 (15.83, 24.48)		56.39 (42.56, 74.72)	
No	85.03 (57.46, 125.84)		336.35 (232.84, 485.88)		20.28 (16.41, 25.05)		54.26 (41.14, 71.56)	
**Mumps**		0.378		0.231		0.806		0.332
Yes	30.00 (30.00, 30.00)		810.00 (7.17, 91489.69)		17.61 (0.01, 23350.11)		89.21 (12.70, 626.50)	
No	78.66 (59.15, 104.61)		334.74 (259.35, 432.05)		20.08 (17.24, 23.40)		54.40 (44.63, 66.31)	
**Rabies**		0.120		0.939		0.429		0.676
Yes	67.14 (33.09, 136.23)		350.72 (194.48, 632.50)		17.41 (12.07, 25.12)		41.54 (26.89, 64.19)	
No	79.09 (57.92, 108.00)		342.39 (256.29, 457.43)		20.56 (17.38, 24.31)		59.92 (48.19, 74.52)	

Data are mean (95% CI). Comparisons were performed using the Student t-test, unless marked as an ANOVA test. ^*^Significant difference (p-value < 0.05). GMT, geometric mean titer.

### 3.3 Analysis of breakthrough infection

#### 3.3.1 Breakthrough infection in the two vaccine groups

**Figure 3:**
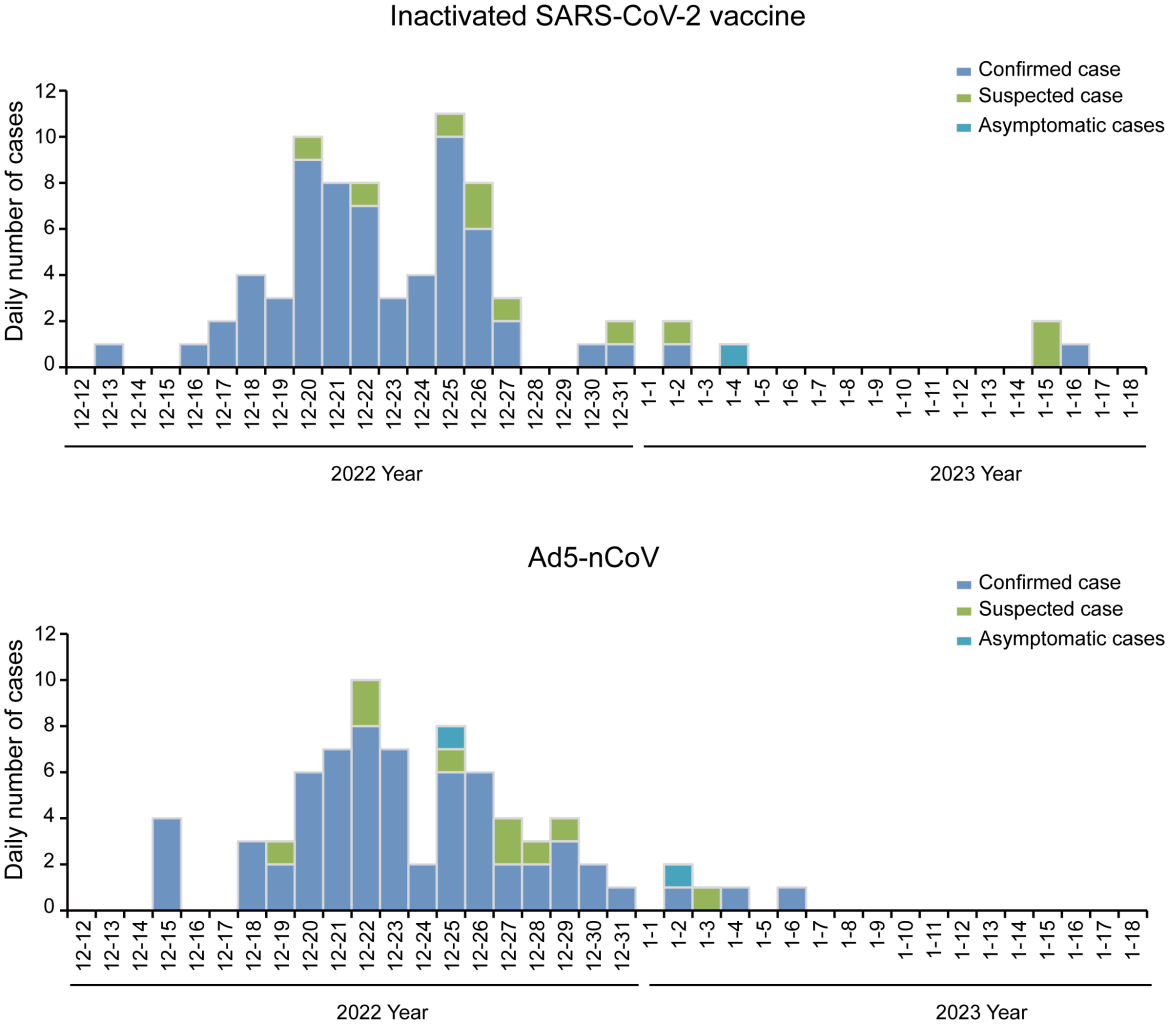
Epidemiologic curve of breakthrough cases. Daily numbers of confirmed cases, suspected cases, and asymptomatic cases. In March 2023, the participants were followed up by telephone to collect information on breakthrough infection. The breakthrough infection occurred from 13 December 2022 to 16 January 2023. Peak occurred between 20 December 2022 and 26 December 2022, counted 47.12% (90/191) during this time.

**Figure 4:**
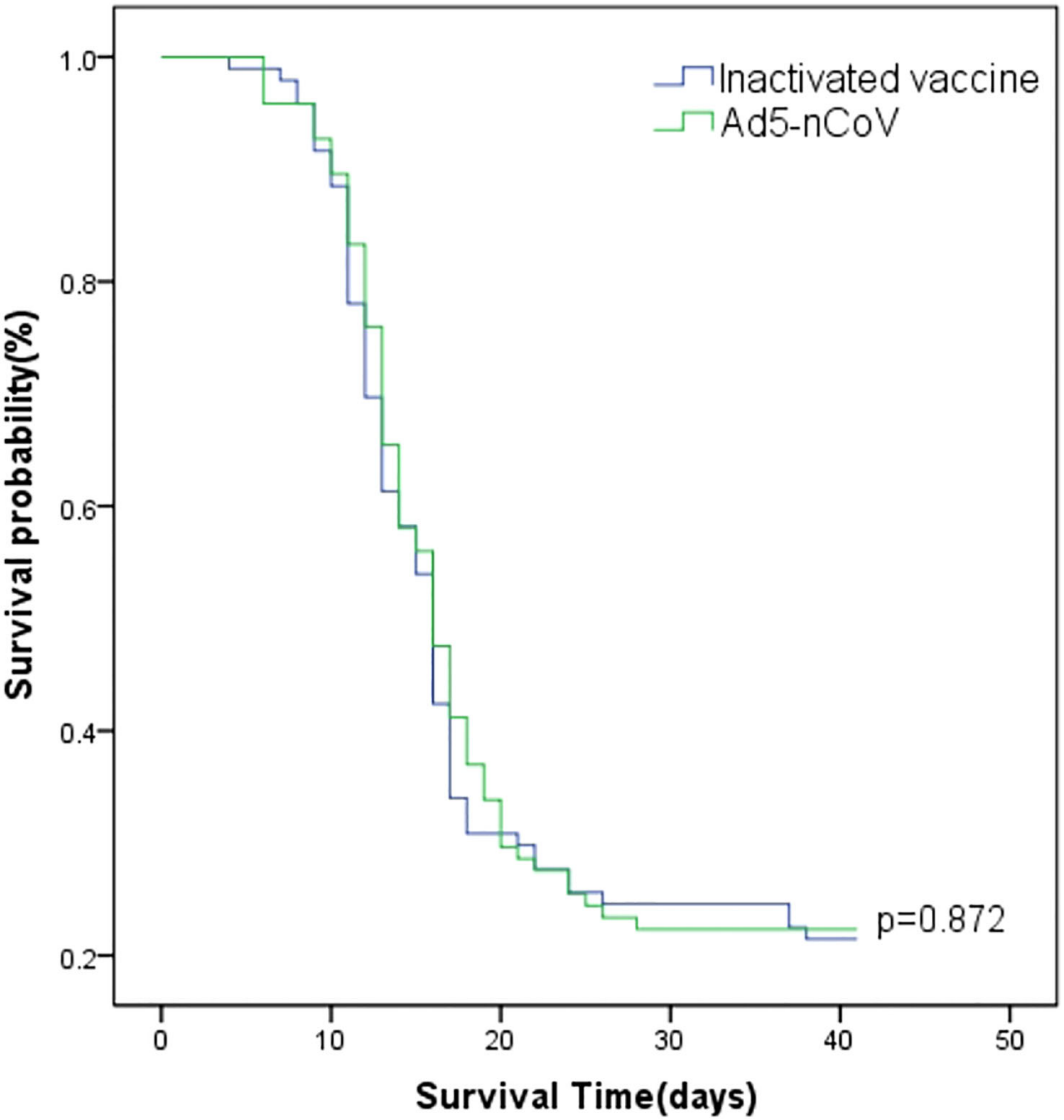
Survival curves of breakthrough infection. The Cox model estimate shows the cumulative probability of breakthrough infection by vaccination type adjusted for sex, age, chronic diseases, and influenza vaccine history, starting on 12 December 2022.

Between 13 December 2022 and 16 January 2023, 66.49% (127/191) of participants developed symptoms and had positive nucleic acid or antigen test results, 9.95% (19/191) of participants developed symptoms and had suspected infection without nucleic acid or antigen test results, 1.57% (3/191) of participants were asymptomatic and had positive nucleic acid or antigen test results, and 21.99% (42/191) were asymptomatic with or without nucleic acid or antigen test results. The breakthrough infection peak occurred between 20 December 2022 and 26 December 2022 counted 47.12% (90/191) during this time (**Figure 3**). The breakthrough infection rates for the Ad5-nCoV and the inactivated vaccine groups were 77.89% and 78.13%, respectively. Survival curve analysis adjusted for sex, age, chronic diseases, and influenza vaccine history showed no statistically significant difference in breakthrough infection between the two groups (*p =* 0.872; **Figure 4**).

**Figure 5:**
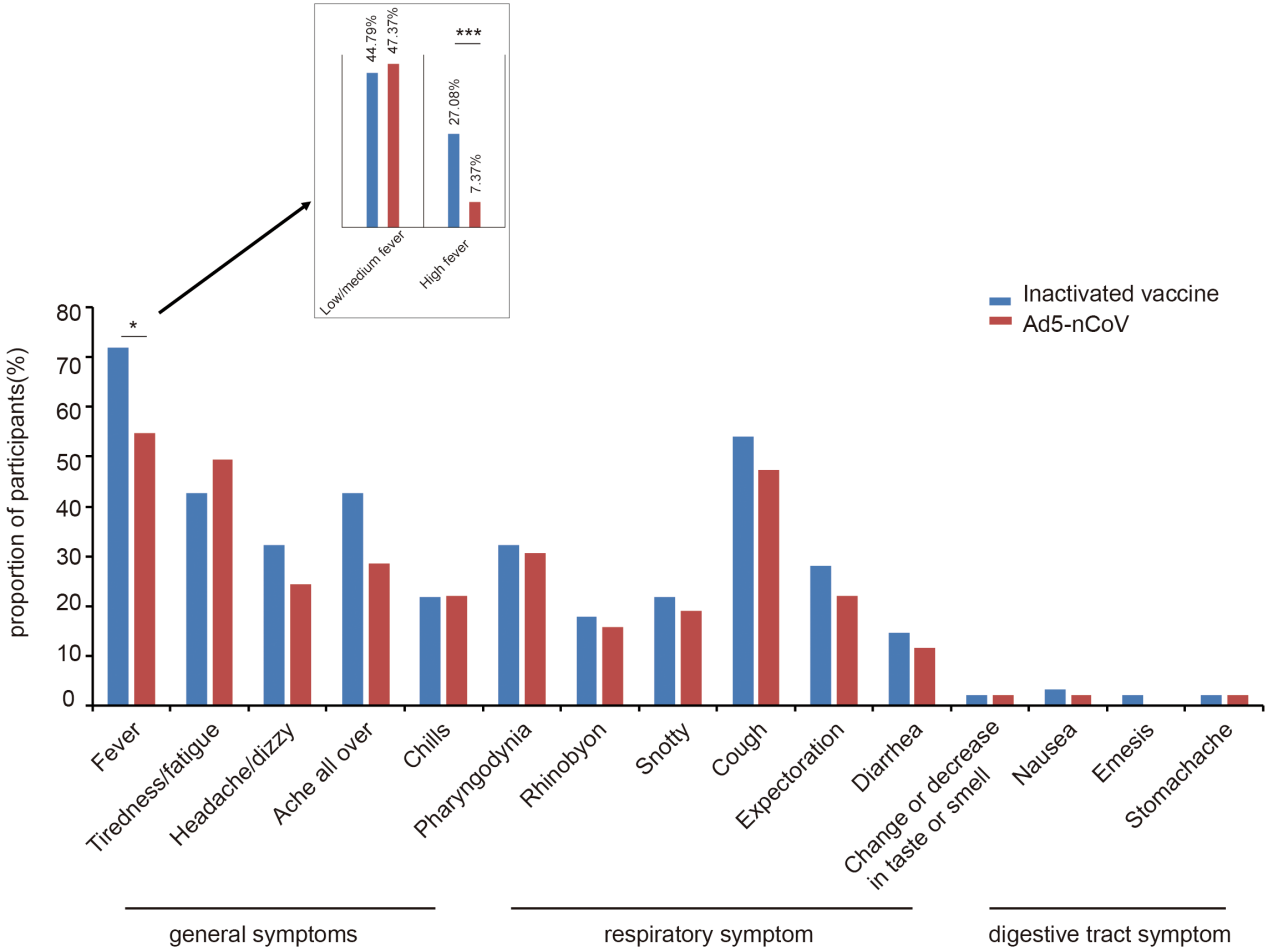
Breakthrough symptoms of the two vaccine groups. The incidence of fever in the Ad5-nCoV group (54.74%) was lower than that in the inactivated vaccine group (71.88%, *p* = 0.014); the incidence of high fever especially was lower in the Ad5-nCoV group (7.37%) than that in the inactivated vaccine (27.08%, *p* < 0.001). *p < 0.05; ***p < 0.001.

In total, 14.14% (27/191) of participants visited outpatient clinics for treatment and 50.26% (96/191) of participants significantly recovered from symptoms within 3 days. However, 1.05% (2/191) of participants (female, inactivated vaccine booster) had symptoms from which they had not significantly recovered more than 2 months after infection. No statistical differences were observed in the incidence of COVID-19 (*p* = 0.952), medical treatment situation (*p* = 0.601), or symptom recovery time (*p* = 0.784) between the two vaccine groups (**Table 3**). The most common symptom among all the participants was fever (63.35%, 121/191), followed by cough (50.79%, 97/191; **Figure 5**). Among all participants, 56.54% (108/191) had at least one of the general symptoms of tiredness/fatigue, headache/dizziness, overall aches, or chills; 60.73% (116/191) had at least one of the general symptoms of pharyngodynia, rhinobyon, nasal mucus discharge, cough, or expectoration; and 17.28% (33/191) had at least one of the digestive tract symptoms of change or decrease in taste or smell, diarrhea, nausea, emesis, or stomachache. No statistical difference in symptoms other than fever was observed between the two vaccine groups. The incidence of fever in the Ad5-nCoV group (54.74%) was lower than that in the inactivated vaccine group (71.88%, *p* = 0.014); the incidence of high fever especially was lower in the treatment (7.37%) versus the control 27.08%) group (*p* < 0.001; **Table 3**).

**Table 3:** The basic profile and symptoms of breakthrough infection.

Variable	Inactivated vaccine (n = 96)	Ad5-nCoV (n = 95)	*p*
Breakthrough cases			0.953^a^
Confirmed cases	64 (66.67)	63 (66.32)	
Suspected cases	10 (10.42)	9 (9.47)	
Asymptomatic cases	1 (1.04)	2 (2.11)	
Uninfection^#^	21 (21.88)	21 (22.11)	
Medical treatment situation			0.601
Asymptomatic	22 (22.92)	23 (24.21)	
Symptomatic with no need	58 (60.42)	61 (64.21)	
Symptomatic with ambulatory treatment	16 (16.67)	11 (11.58)	
Symptom recovery time			
Asymptomatic	22 (22.92)	23 (24.21)	0.784^a^
<3 days	26 (27.08)	25 (26.32)	
3–5 days	21 (21.88)	18 (18.95)	
6–7 days	16 (16.67)	15 (15.79)	
8–14 days	7 (7.29)	9 (9.47)	
>14 days	2 (2.08)	5 (5.26)	
Not yet improved	2 (2.08)	0 (0.00)	
Symptoms			
Fever			0.001*
No	27 (28.13)	43 (45.26)	
Low/medium fever (37.4°C–39°C)	43 (44.79)	45 (47.37)	
High fever (≥39.1°C)	26 (27.08)	7 (7.37)	
General symptom			0.934
No	42 (43.75)	41 (43.16)	
Yes	54 (56.25)	54 (56.84)	
Respiratory symptom			0.424
No	35 (36.46)	40 (42.11)	
Yes	61 (63.54)	55 (57.89)	
Digestive tract symptom			0.356
No	77 (80.21)	81 (85.26)	
Yes	19 (19.79)	14 (14.74)	

The “uninfection^#^” group contains participants who were asymptomatic and did not undergo nucleic acid or antigen testing or were asymptomatic and had negative nucleic acid or antigen test results. Data are number of participants (%). Comparisons were performed using Chi-squared test, unless marked as ^a^Fisher’s exact test. *Significant difference (p-value < 0.05).

#### 3.3.2 Factors influencing breakthrough infection

Participants aged 18–59 years in the inactivated vaccine group were more likely to have digestive tract symptoms compared with who aged ≥60 years (*p* = 0.011; **Table 4**). Those who exercised ≥3 h per week in the Ad5-nCoV group and participants with a history of influenza vaccine uptake in the inactivated vaccine group recovered significantly faster than who exercised <3 h per week (*p* = 0.035 and *p* = 0.038; **Table 5**).

**Table 4:** Associations between participants’ characteristics and the breakthrough infection.

Variable	Breakthrough infection				Medical treatment situation				Symptom recovery time			
	Inactivated vaccine (n = 96)		Ad5-nCov (n = 95)		Inactivated vaccine (n = 96)		Ad5-nCov (n = 95)		Inactivated vaccine (n = 96)		Ad5-nCov (n = 95)	
	Infection rate (%)	*p*	Infection rate (%)	*p*	Need ambulatory treatment (%)	*p*	Need ambulatory treatment (%)	*p*	≥3 days (%)	*p*	≥3 days (%)	*p*
**Gender**		0.555		0.335		1.000		0.205		0.100		0.052
Male	75.93		74.00		16.67		16.67		42.59		40.00	
Female	80.95		82.22		16.67		16.67		59.52		60.00	
**Age, years**		0.900		0.415		0.711		0.516^a^		0.214		0.589
18–59	78.57		75.00		17.86		14.29		55.36		51.79	
≥60	77.50		82.05		15.00		7.69		42.50		46.15	
**BMI(kg/m^2^)**		0.249^a^		0.525^a^		0.826^a^		1.000^a^		0.419^a^		0.317^a^
<18.5	100.00		75.00		0.00		0.00		0.00		75.00	
18.5–23.9	70.37		82.22		16.67		13.33		44.44		55.56	
24.0–27.9	86.67		69.70		20.00		12.12		60.00		45.45	
≥28.0	90.91		84.62		9.09		7.69		54.55		30.77	
**Exercise**		0.772		0.596		0.340		1.000^a^		0.200		0.035*
≥3 h per week	79.03		76.47		19.35		11.76		54.84		42.65	
<3 h per week	76.47		81.48		11.76		11.11		41.18		66.67	
**Sleep**		0.559^a^		0.889		0.757		0.486		0.331		0.950
Regular	79.73		78.26		16.22		16.22		47.30		49.28	
Have insomnia	72.73		76.92		18.18		18.18		59.09		50.00	
**Underlying chronic diseases**		0.765		0.653		1.000^a^		1.000^a^		0.816		0.772
Yes	78.87		76.56		16.90		12.50		49.30		48.44	
No	76.00		80.65		16.00		9.68		52.00		51.61	
Vaccination history												
**Influenza**		0.260		0.639		0.459		0.073		0.038*		0.125
Yes	72.50		80.00		20.00		17.78		37.50		57.78	
No	82.14		76.00		14.29		6.00		58.93		42.00	
**Mumps**		1.000^a^		1.000^a^		0.307^a^		1.000^a^		1.000^a^		0.117^a^
Yes	100.00		100.00		50.00		0.00		50.00		100.00	
No	77.66		77.17		15.96		11.96		50.00		47.83	
**Rabies**				0.775^a^		1.000^a^		0.061		0.160		0.763
Yes	86.67	0.510^a^	76.19		13.33		23.81		66.67		52.38	
No	76.54		78.38		17.28		8.11		46.91		48.65	

Data are number of participants (%).Comparisons were performed using the Chi-squared test, unless marked as ^a^ Fisher’s exact test. ^*^Significant difference (p-value < 0.05). BMI, body mass index.

**Table 5:** Associations between participants’ characteristics and the symptoms of breakthrough cases.

Variable	Fever				General symptom				Respiratory symptom				Digestive tract symptom			
	Inactivated vaccine (n = 96)		Ad5-nCov (n = 95)		Inactivated vaccine (n = 96)		Ad5-nCov (n = 95)		Inactivated vaccine (n = 96)		Ad5-nCov (n = 95)		Inactivated vaccine (n = 96)		Ad5-nCov (n = 95)	
	%	*p*	%	*p*	%	*p*	%	*p*	%	*p*	%	*p*	%	*p*	%	*p*
**Gender**		0.081		0.071		0.070		0.067		0.894		0.100		0.872		0.428
Male	64.81		46.00		48.15		48.00		62.96		50.00		20.37		12.00	
Female	80.95		64.44		66.67		66.67		64.29		66.67		19.05		17.78	
**Age, years**		0.205		0.572		0.060		0.301		0.299		0.505		0.011*		0.660
18–59	76.79		57.14		64.29		60.71		67.86		67.86		28.57		16.07	
≥60	65.00		51.28		45.00		51.28		57.50		57.50		7.50		12.82	
**BMI (kg/m^2^)**		0.423^a^		0.781^a^		0.585^a^		0.841^a^		0.100^a^		0.863^a^		0.689^a^		0.557^a^
< 18.5	100.00		50.00		0.00		75.00		0.00		50.00		0.00		0.00	
18.5–23.9	66.67		60.00		59.26		57.78		55.56		62.22		16.67		11.11	
24.0–27.9	73.33		48.48		50.00		51.52		76.67		54.55		23.33		18.18	
≥28.0	90.91		53.85		63.64		61.54		72.73		53.85		27.27		23.08	
**Exercise**		0.789		0.577		0.420		0.223		0.861		0.121		0.224		0.531^a^
≥3 h per week	70.97		52.94		53.23		52.94		62.90		52.94		16.13		13.24	
<3 h per week	73.53		59.26		61.76		66.67		64.71		70.37		26.47		18.52	
**Sleep**		0.919		0.414		0.760		0.409		0.621		0.364		0.131^a^		0.751^a^
Regular	71.62		52.17		55.41		59.42		64.86		64.86		16.22		15.94	
Have insomnia	72.73		61.54		59.09		50.00		59.09		59.09		31.82		11.54	
**Underlying chronic diseases**		0.987		0.989		0.618		0.474		0.956		0.338		1.000^a^		0.767^a^
Yes	71.83		54.69		57.75		59.38		63.38		60.94		19.72		14.06	
No	72.00		54.84		52.00		51.61		64.00		51.61		20.00		16.13	
Vaccination history																
**Influenza**		0.420		0.071		0.835		0.555		0.858		0.100		0.574		0.051
Yes	67.50		64.44		55.00		60.00		62.50		62.50		22.50		22.22	
No	75.00		46.00		57.14		54.00		64.29		64.29		17.86		8.00	
**Mumps**		1.000^a^		1.000^a^		0.189^a^		0.256^a^		0.130^a^		1.000^a^		1.000^a^		1.000^a^
Yes	100.00		66.67		0.00		100.00		0.00		66.67		0.00		0.00	
No	71.28		54.35		57.45		55.43		64.89		57.61		20.21		15.22	
**Rabies**		0.545		0.215		0.376		0.975		0.391		0.280		0.488^a^		0.728^a^
Yes	80.00		42.86		66.67		57.14		73.33		47.62		26.67		9.52	
No	70.37		58.11		54.32		56.76		61.73		60.81		18.52		16.22	

Comparisons were performed using the Chi-squared test unless marked as ^a^Fisher’s exact test. ^*^Significant difference (p-value < 0.05).

A multivariable logistic regression analysis was performed to test the adjusted association between the main independent variable, defined as the type (inactivated vaccine or Ad5-nCoV) of the fourth vaccine dose vaccine and 1) the seropositivity rate of anti-RBD IgG and neutralizing antibodies against the BA.4/5 pseudovirus and 2) the risk of breakthrough infection with SARS-CoV-2. The association was tested after adjustment for sex, age, body mass index, exercise, sleep, underlying chronic disease, and influenza vaccination history.

Compared with the participants who received the inactivated vaccine as the fourth dose, the participants who received Ad5-nCoV experienced an increase in the seropositivity rate of anti-RBD IgG [relative risk (RR) = 8.58; 95% CI, 3.53–20.86] and neutralizing antibodies against the BA.4/5 pseudovirus (RR = 24.52; 95% CI, 6.62–90.87) at 6 months after the last vaccination and were at a lower risk of fever caused by SARS-CoV-2 (RR = 0.41; 95% CI, 0.22–0.78). Compared with their male counterparts, female participants were at a higher risk of fever (RR = 2.44; 95% CI, 1.23–4.83) and general symptoms (RR = 2.44; 95% CI, 1.06–3.87) caused by SARS-CoV-2 and at a higher risk of symptom recovery time ≥3 days (RR = 2.45; 95% CI, 1.27–4.70). Compared with participants aged 18–59 years, participants aged ≥60 years had a lower seropositivity rate of neutralizing antibodies against BA.4/5 pseudovirus (RR = 0.18; 95% CI, 0.05–0.64) and were at a lower risk of digestive tract symptoms (RR = 0.29; 95% CI, 0.10–0.85). Compared with participants with a body mass index <24.0 kg/m^2^, participants with a body mass index ≥24.0 kg/m^2^ had a higher risk of digestive tract symptoms (RR = 2.82; 95% CI, 1.12–7.14; **Table 6**).

**Table 6:** Multifactorial analysis of antibody levels and breakthrough infection.

Variable	Anti–RBD-IgG		Neutralizing antibodies to Pseudovirus (BA.4/5)		Breakthrough infection		Medical treatment situation		Symptom recovery time		Fever		General symptom		Respiratory symptom		Digestive tract symptom	
	RR	*p*	RR	*p*	RR	*p*	RR	*p*	RR	*p*	RR	*p*	RR	*p*	RR	*p*	RR	*p*
Vaccine type (Ad5-nCov vs. Inactivated vaccine)																		
	8.58 (3.53–20.86)	<0.001*	24.52 (6.62–90.87)	<0.001*	0.98 (0.49–1.97)	0.956	0.56 (0.23–1.32)	0.183	0.90 (0.49–1.62)	0.715	0.41 (0.22–0.78)	0.006*	1.01 (0.56–1.84)	0.964	0.75 (0.41–1.37)	0.346	0.6 (0.27–1.34)	0.211
Gender (Female vs. Male)																		
	1.94 (0.81–4.62)	0.135	0.94 (0.37–2.40)	0.892	1.81 (0.83–3.93)	0.135	1.37 (0.55–3.44)	0.502	2.45 (1.27–4.70)	0.007*	2.44 (1.23–4.83)	0.011*	2.03 (1.06–3.87)	0.032	1.59 (0.83–3.06)	0.163	1.45 (0.59–3.55)	0.416
Age (≥60 years vs. 18–59 years)																		
	0.66 (0.28–1.54)	0.336	0.18 (0.05–0.64)	0.008*	1.28 (0.56–2.93)	0.564	0.67 (0.25–1.85)	0.444	0.59 (0.29–1.20)	0.147	0.66 (0.32–1.38)	0.274	0.62 (0.31–1.25)	0.179	0.79 (0.39–1.59)	0.504	0.29 (0.10–0.85)	0.024*
BMI (≥24.0 kg/m^2^ vs. <24.0 kg/m^2^)																		
	1.74 (0.73–4.14)	0.208	0.57 (0.22–1.50)	0.258	1.52 (0.70–3.30)	0.292	1.21 (0.47–3.07)	0.693	1.39 (0.72–2.70)	0.332	1.51 (0.76–3.01)	0.241	1.09 (0.57–2.11)	0.79	1.83 (0.93–3.59)	0.078	2.82 (1.12–7.14)	0.028*
Exercise (< 3 h per week vs. ≥3 h per week)																		
	0.74 (0.32–1.75)	0.499	2.28 (0.88–5.92)	0.090	1.14 (0.51–2.57)	0.743	0.46 (0.17–1.28)	0.138	1.01 (0.51–1.98)	0.976	0.98 (0.48–2.01)	0.959	1.38 (0.69–2.74)	0.363	1.30 (0.65–2.60)	0.466	1.38 (0.58–3.29)	0.471
Sleep (Have insomnia vs. Regular)																		
	1.78 (0.68–4.65)	0.240	1.15 (0.41–3.22)	0.795	0.88 (0.38–2.04)	0.768	1.41 (0.52–3.77)	0.498	1.41 (0.68–2.92)	0.350	1.34 (0.61–2.90)	0.465	0.77 (0.37–1.58)	0.471	1.12 (0.54–2.34)	0.761	1.50 (0.59–3.81)	0.393
Underlying chronic diseases (Yes vs. No)																		
	1.02 (0.38–2.71)	0.969	1.37 (0.37–5.08)	0.633	1.02 (0.40–2.58)	0.966	0.76 (0.24–2.34)	0.626	1.86 (0.84–4.12)	0.124	1.35 (0.6–3.04)	0.474	1.16 (0.54–2.53)	0.702	0.88 (0.40–1.93)	0.749	1.75 (0.58–5.28)	0.317
Influenza vaccination history (No vs. Yes)																		
	1.49 (0.67–3.31)	0.326	1.40 (0.54–3.63)	0.494	1.20 (0.57–2.52)	0.639	0.44 (0.18–1.09)	0.076	1.44 (0.76–2.71)	0.264	0.91 (0.47–1.77)	0.788	1.01 (0.54–1.90)	0.973	0.75 (0.40–1.42)	0.380	0.65 (0.28–1.52)	0.321

Data are RR (95% confidence interval). Binary logistic regression was used for multifactorial analysis.^*^Significant difference (p-value < 0.05). RR, relative risk.

## 4 Discussion

Neutralizing antibodies are reported to act as a correlate of protection against COVID-19; therefore, a boost in these antibodies suggests an induced response associated with vaccine efficacy (24). Previous studies have shown that in individuals previously vaccinated with three doses of inactivated SARS-CoV-2 vaccine, heterologous regimens with intramuscular Ad5-nCoV induced significantly higher titers of neutralizing antibodies against both wild-type SARS-CoV-2 and the BA.4/5 pseudovirus at days 14 and 28 after vaccination than the homologous booster schedule with inactivated virus (20). The findings of our 10-month randomized, parallel-controlled prospective cohort study further indicated that the second heterologous booster with intramuscular Ad5-nCoV induced higher titers and seropositivity rates of anti-RBD IgG and neutralizing antibodies against the BA.4/5 pseudovirus than the second homologous booster with inactivated vaccine at 6 months after vaccination. Similarly, the third dose (first booster) of the heterologous vaccine with Ad5-nCoV induced a higher antibody response than the homologous vaccines (18, 19, 25, 26), indicating that the heterologous booster regimens containing Ad5-nCoV were superior to the homologous schedule with regard to the first and second boosters (third and fourth doses). Similar results have been reported that heterologous boosting resulted in more robust immune responses than homologous boosting with other COVID-19 vaccines such as mRNA-1273 (Moderna), BNT162b2 (Pfizer–BioNTech), AZD1222 (Astra Zeneca), (Ad26.COV2-S, Janssen), and ChAdOx1 nCoV-19 vaccine (AZD1222, AstraZeneca) (27–31).

The overall infection rate during the nationwide COVID-19 pandemic that followed the Chinese government’s “New ten measures” policy issued on 7 December 2022 (21) was estimated at 87.54% (22). By following participants who received a fourth-dose vaccination with Ad5-nCoV or the inactivated SARS-CoV-2 vaccine, we observed that 78.01% (149/191) of participants were infected or suspected to be infected with SARS-CoV-2. The breakthrough infection peak occurred between 20 December 2022 and 26 December 2022, consistent with the nationwide peak on 22 December 2022 (23). Furthermore, no differences were observed between the Ad5-nCoV booster group and the inactivated SARS-CoV-2 vaccine booster group in breakthrough infection rate, medical treatment situation, or symptom recovery time. However, to the best of our knowledge, ours was the first study to report that the fourth dose of Ad5-nCoV is associated with a lower incidence SARS-CoV-2–related fever than the inactivated vaccine booster, indicating that the heterologous booster regimen may moderately alleviate some symptoms of infection.

In addition, our study found that compared with male participants, female participants were at an increased risk of fever and general symptoms caused by SARS-CoV-2 and at a greater risk of symptom recovery time ≥3 days, supporting previous findings that the female sex is a risk factor for long COVID-19 (32). Vaccine responses are widely reported to be weaker in older adults, who experience immunosenescence and a more rapid waning of antibodies than younger people (33–35). We further observed that participants aged ≥60 years had a lower seropositivity rate of neutralizing antibodies against the BA.4/5 pseudovirus versus participants aged 18–59 years. However, no statistical difference in breakthrough infection was observed between the age groups (18–59 vs. ≥60 years). Higher age and body mass index were associated with an increased risk of digestive tract symptoms in our study. Digestive system involvement may protect patients with mild and moderate symptoms from lymphocyte depletion caused by SARS-CoV-2 (36); however, the relevant mechanism of action remains unclear.

Our study has several limitations. First, only adults with stable medical conditions over 18 years of age were recruited; the inclusion of people who require additional protection against COVID-19 such as immunocompromised individuals may have produced more marked results than those observed. Therefore, the recruitment criteria prevented our results from being representative of the general population. Second, because blank controls (i.e., participants who do not receive a fourth vaccination dose) were not recruited into the study, we could not assess the efficacy of a four-dose immunization regimen against breakthrough infection with SARS-CoV-2. Therefore, the protective effect of the second heterologous booster vaccination regimen remains uncertain. Third, the sample size was too small to investigate the number of potentially severe and fatal cases caused by SARS-CoV-2. A larger sample size would have increased statistical power to allow the identification of influencing factors among subpopulations. Fourth, information on breakthrough infection in the follow-up was based only on participant recall, introducing potential recall bias. Finally, live virus neutralization antibodies against wild-type SARS-CoV-2, Omicron BA.4/5, or other current Omicron subvariants such as BF.7, BQ.1, and XBB that could increase the generalizability of immunogenicity results were not used in our study and must be further explored.

In conclusion, a heterologous fourth dose with Ad5-nCoV caused higher antibody levels than a homologous fourth dose with the inactivated SARS-CoV-2 vaccine at 6 months after the last vaccination and decreased the risk of fever caused by SARS-CoV-2 in healthy adults who had been immunized with three doses of the inactivated vaccine. However, the two vaccine types showed equivalent efficacy after the pandemic. Our findings support the heterologous administration of Ad5-nCoV over the homologous administration of the inactivated SARS-CoV-2 vaccine. Furthermore, next-generation vaccines may be needed to provide better protection against COVID-19 by addressing the immune escape of SARS-CoV-2 variants.

## Data availability statement

The raw data supporting the conclusions of this article will be made available by the authors, without undue reservation.

## Ethics statement

The studies involving humans were approved by The Research Ethics Committee of the Zhejiang Provincial Center of Disease Control and Prevention (ethics code number: 2022-021-01). The studies were conducted in accordance with the local legislation and institutional requirements. The participants provided their written informed consent to participate in this study.

## Author contributions

XH, HL, JJ, LZ, and HZ were the principal investigators who designed the research and coordinated the study; NX, PQ, YY, and RD led and participated in the site work, including the recruitment, follow-up, and data collection; PW and YX supervised the study; HZ was responsible for laboratory analyses; NX and HZ did the statistical analysis and wrote the manuscript. All authors contributed to the article and approved the submitted version.
