# Supplementary material for: Study of efficacy and antibody duration to fourth-dose booster of Ad5-nCoV or inactivated SARS-CoV-2 vaccine in Chinese adults: a prospective cohort study
Source: Front Immunol. 2023 Sep 6;14:1244373. doi: 10.3389/fimmu.2023.1244373 (PMC10510200; doi:10.3389/fimmu.2023.1244373)
Supplement: Supplementary file 1 [file Table_1.docx]

Supplementary Material

Study of efficacy and antibody duration to fourth dose booster of Ad5-nCoV or inactivated SARS-CoV-2 vaccine in Chinese adults: A prospective cohort study

Nani Xu^1†^, Yu Xu^2†^, Rongrong Dai^3†^, Pan Qin^1^, Peng Wan^2^, Zheng Lin^1^, Yejing Yang^1^, Jianmin Jiang^4,5^, Hangjie Zhang^4,5*^, Xiaowei Hu^1*^, Huakun Lv^4,5*^

# Supplementary Table

**Supplementary table 1** SARS-CoV-2 RBD-specific IgG antibodies and pseudovirus-neutralizing antibodies to Omicron BA.4/5 at different times of two groups

| **Variable** | **Before vaccination** | | | **6 monthes after vaccination** | | |
| --- | --- | --- | --- | --- | --- | --- |
|  | **Inactivated vaccine(n=96)** | **Ad5-nCoV (n=95)** | ***p*** | **Inactivated vaccine(n=96)** | **Ad5-nCoV (n=95)** | ***p*** |
| **Anti-RBD-IgG** | | | | | |  |
| GMT | 59.76 | 63.89 | 0.733 | 77.10 | 344.22 | ＜0.001* |
|  | (44.68-79.93) | (49.37-82.67) |  | (58.24-102.05) | (266.65-444.35) |  |
| **Neutralizing antibodies to Pseudovirus (BA.4/5)** | | | | | |  |
| GMT | 33.75 | 29.71 | 0.073 | 20.03 | 55.26 | ＜0.001* |
|  | (30.65-37.17) | (26.82-32.91) |  | (17.23-23.28) | (45.52-67.08) |  |

Data are mean (95% CI) or participants(95% CI). The comparison was analyzed by T test unless it is marked with a Chi-squared test, b Fisher’s exact test.^*^Significant difference (*p*-value < 0.05).

**
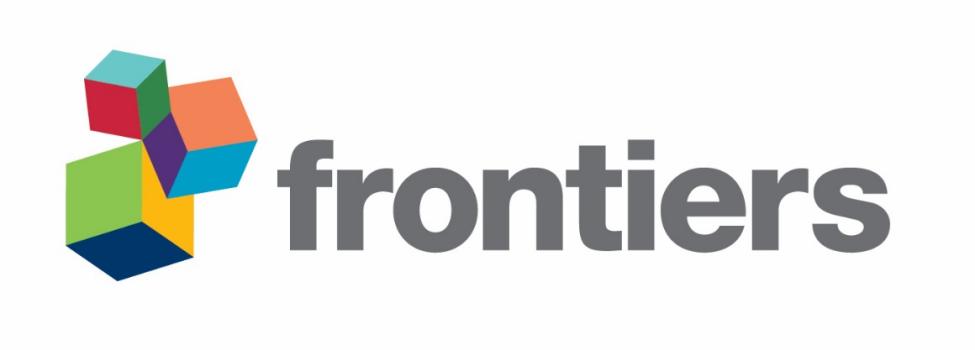
**
